# Prolonged Instability Prior to a Regime Shift

**DOI:** 10.1371/journal.pone.0108936

**Published:** 2014-10-03

**Authors:** Trisha L. Spanbauer, Craig R. Allen, David G. Angeler, Tarsha Eason, Sherilyn C. Fritz, Ahjond S. Garmestani, Kirsty L. Nash, Jeffery R. Stone

**Affiliations:** 1 Department of Earth and Atmospheric Sciences and School of Biological Sciences, University of Nebraska–Lincoln, Lincoln, Nebraska, United States of America; 2 U.S. Geological Survey, Nebraska Cooperative Fish and Wildlife Research Unit, School of Natural Resources, University of Nebraska–Lincoln, Lincoln, Nebraska, United States of America; 3 Department of Aquatic Sciences and Assessment, Swedish University of Agricultural Sciences, Uppsala, Sweden; 4 Office of Research and Development, National Risk Management Research Laboratory, U.S. Environmental Protection Agency, Cincinnati, Ohio, United States of America; 5 Australian Research Council Centre of Excellence for Coral Reef Studies, James Cook University, Townsville, Queensland, Australia; 6 Department of Earth and Environmental Systems, Indiana State University, Terre Haute, Indiana, United States of America; University of Cambridge, United Kingdom

## Abstract

Regime shifts are generally defined as the point of ‘abrupt’ change in the state of a system. However, a seemingly abrupt transition can be the product of a system reorganization that has been ongoing much longer than is evident in statistical analysis of a single component of the system. Using both univariate and multivariate statistical methods, we tested a long-term high-resolution paleoecological dataset with a known change in species assemblage for a regime shift. Analysis of this dataset with Fisher Information and multivariate time series modeling showed that there was a∼2000 year period of instability prior to the regime shift. This period of instability and the subsequent regime shift coincide with regional climate change, indicating that the system is undergoing extrinsic forcing. Paleoecological records offer a unique opportunity to test tools for the detection of thresholds and stable-states, and thus to examine the long-term stability of ecosystems over periods of multiple millennia.

## Introduction

Ecosystems can undergo regime shifts and reorganize into an alternative state when a critical threshold is exceeded [1]–[3]. Most quantitative regime shift research has focused on abrupt shifts that have occurred during a period of human observation; this has resulted in a better understanding of how fast variables (e.g. nutrient loading) erode resilience, but it hasn't addressed how slow variables (e.g. long-term changes in climate) can alter ecosystem state. Paleoecological records can provide insight on the frequency and duration of transitions between alternative states in systems that are affected by both fast and slow variables, at timescales not accessible in the observed record.

To test for regime shifts in the paleoecological record, we used a long-term high-resolution sedimentological record from Foy Lake (Montana, USA) that showed abrupt changes in diatom community structure at ∼1.3 ka (thousands of years before present, with present defined as AD 1950). Foy Lake (48.1648°N, 1143589°W, 1005 m elevation) is a deep freshwater lake situated in the drought-sensitive Flathead River Basin in the Northern Rocky Mountains [4], [5]. Diatom assemblages in this system are sensitive to changes in lake depth driven by changes in effective moisture [6] and represent one metric of ecological resilience. The percent abundances of 109 diatom species were collected from a lake sediment core that was sampled continuously at an interval of every ∼5–20 years, yielding a ∼7 kyr record of 800 time-steps.

To determine if regimes shifts could be anticipated in this paleoecological data set we (i) plotted several indicators proposed to be early-warning signals of approaching critical thresholds (increasing variance, skewed responses, kurtosis, and the autocorrelation at lag-1) [7] against time, (ii) collapsed the 109 species variables into the system's mean Fisher information (FI) [8], and (iii) used multivariate time series modeling based on canonical ordination [9]. Many of these statistical early-warning signals have been developed based on bifurcation theory, and they have successfully anticipated regime shifts in many [10]–[13], but not all [14] systems tested. Increasing variance, skewed responses, and kurtosis in time-series data may be indicative of flickering, the rapid alternating between two different states prior to a regime shift [15]. Along with autocorrelation at lag-1, increasing variance in time-series data can be caused by critical slowing down, where a system is slow to recover from minor disturbances as it approaches a critical transition [7]. These univariate metrics can be limited in their utility, because appreciable signals often occur at the onset of the regime shift, which is generally too late to implement effective management actions [16]. Hence, we sought methods (FI and multivariate time series modeling) that more effectively investigate the dynamics of complex multivariate systems. FI, an integrated index based on information theory, declines as it approaches a regime shift, indicating loss of order and increasing variability, and the regime shift is typically identified as a minimum FI value. Afterward, FI will often increase before settling into a new regime [8]. FI has been used to evaluate stability, regime shifts, and resilience in real complex systems, including ecosystems, climate data, urban systems, and nation states [8], [17]–[25]. Multivariate time series modeling, which models the fluctuation of the frequencies of species or groups of species at distinct temporal scales [9], complements the FI approach. Multivariate time series modeling is sensitive to changes in the abundance and occurrence structure of species in the community. It is capable of identifying scale-specific temporal patterns (fluctuations at scales of decades, centuries, and millennia) in the data and therefore permits assessing how transitional and regime dynamics manifest across the modeled time scales. A key advantage of using these two methods with paleoecological data is that neither requires *a priori* knowledge of system structure or dynamics [8]–[9].

## Results and Discussion

**Figure 1:**
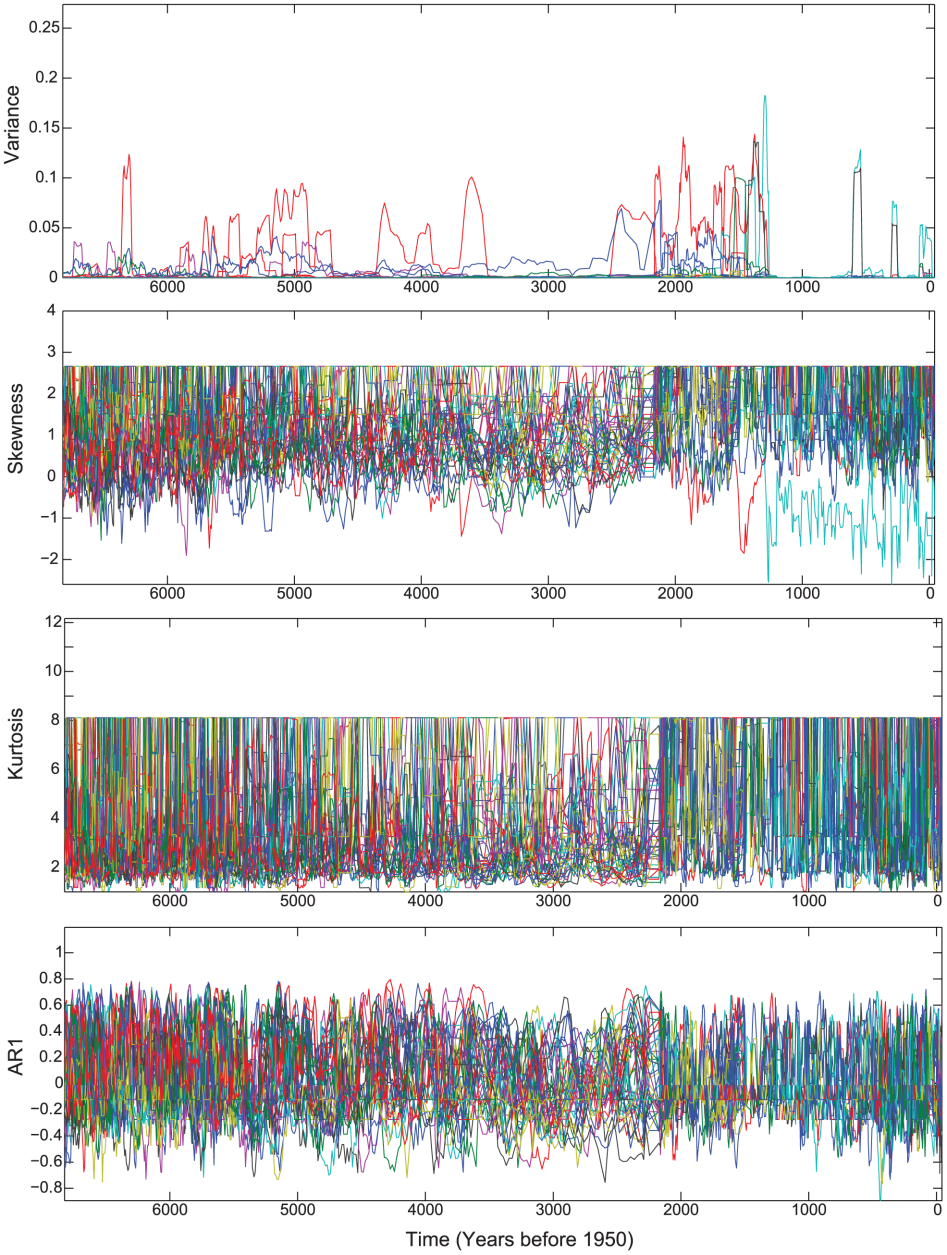
Early warning signals of regime shifts applied to 109 diatom species from Foy Lake. Several populations of species experienced increased variability in the Foy Lake record; this increased variability peaks prior to ∼1.3 ka (**A**). Skewness (**B**), kurtosis (**C**), and critical slowing down (**D**) show no clear trends, although, slight frequency changes can be detected at approximately ∼4.5 ka and ∼2.0 ka.

**Figure 2:**
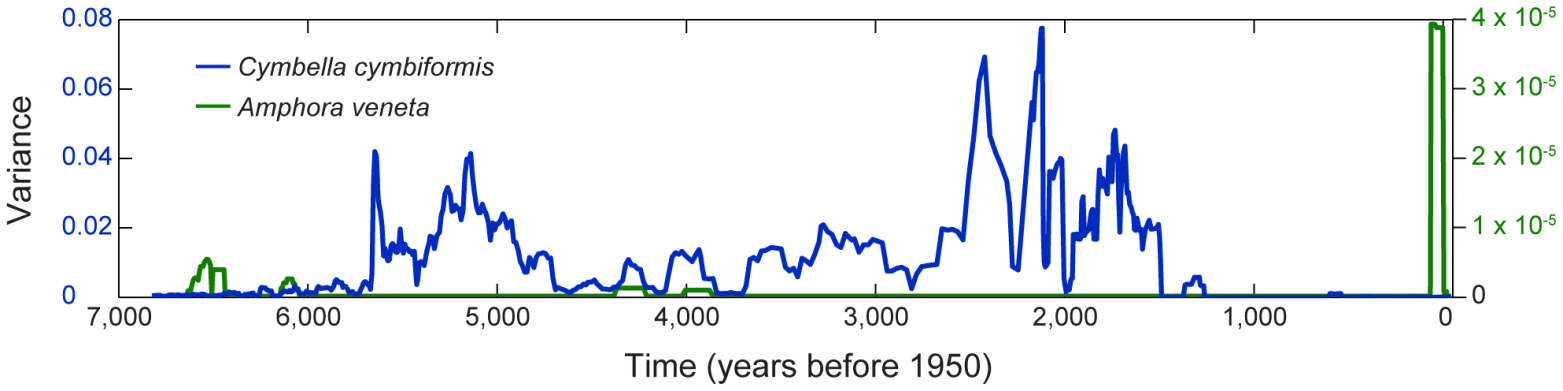
The variance of two diatom species. While *Cymbella cymbiformis* displayed a pattern of increasing variance prior to ∼1.3 ka, *Amphora veneta* did not. Conflicting patterns make it difficult to use univariate statistics to characterize the behavior of a complex multivariate system.

Of the indicators used, we found that univariate species-level indicators were weak predictors of regime shifts. Skewness, kurtosis, and critical slowing down showed minor changes in the frequency patterns of some variables. Several species showed increased variance prior to the abrupt change in species composition at ∼1.3 ka. However, most of the species provided no warning signal; hence, conclusions about the dynamics of the overall system were unclear (Fig. 1). Since indicators must be computed for each variable (i.e., diatom species) individually, characterization of the overall system is difficult [8]. For example, the variance of two diatom species, *Cymbella cymbiformis* and *Amphora veneta*, showed very different patterns in variance (Fig. 2). The former would be a good candidate for anticipating the transition in community structure in Foy Lake, while variance in the latter species was random in relation to large scale community shifts. While some particular species might serve as a leading indicator of a regime shift in this system, it is impossible, *a priori*, to identify which species might be appropriate to monitor. In addition, an early-warning indicator species that is effective in Foy Lake may not be useful in other systems, because of differences in physical, chemical, or biological variables that affect community interactions. In summary, it was difficult to detect a community-level regime shift from any of the traditional indicators of early-warning signals, because of the multivariate nature of the study system and the univariate capacity of indicators.

**Figure 3:**
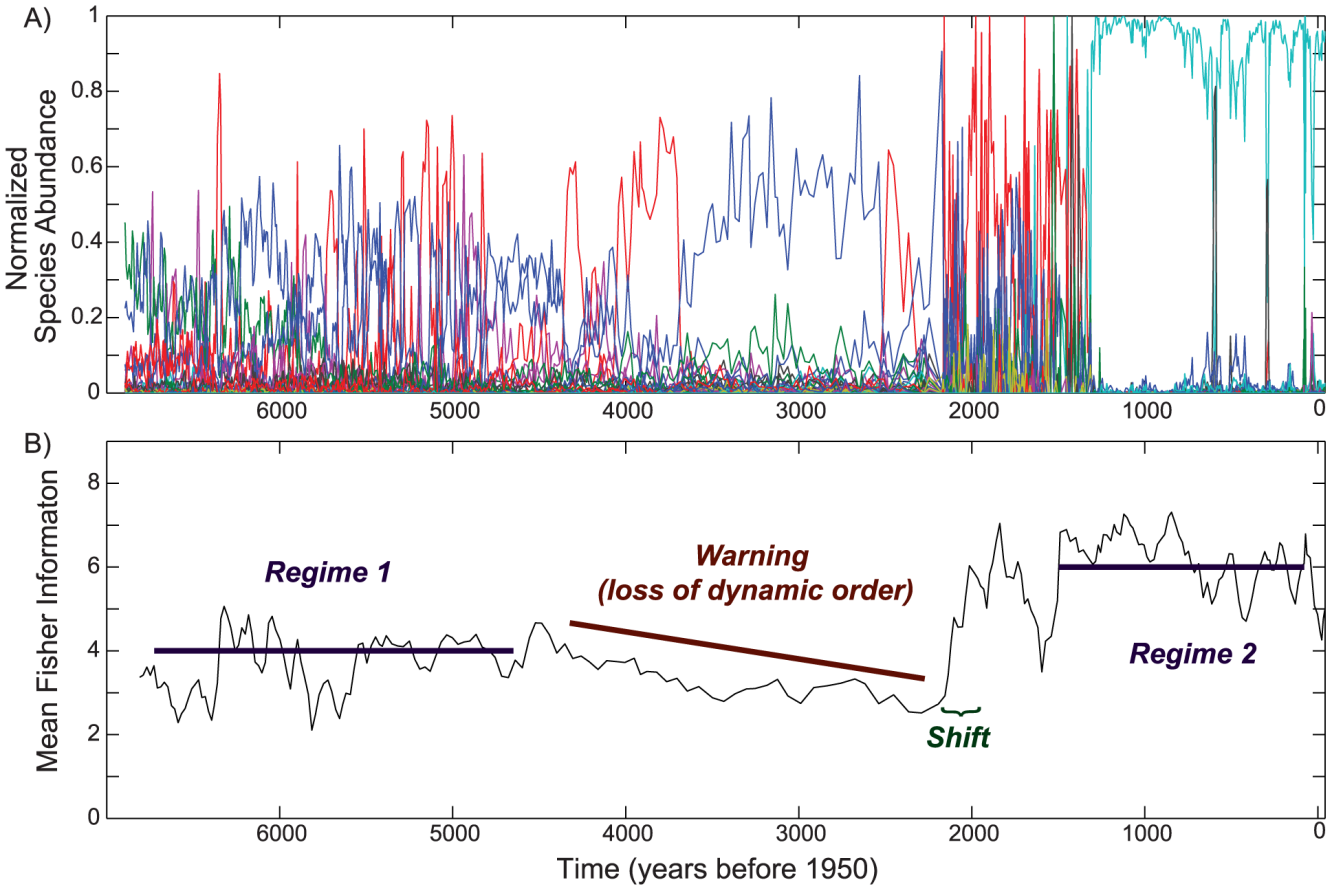
Normalized diatom species abundance for all species (A) and mean Fisher Information (B) for Foy Lake. Prior to ∼4.5 ka the system had episodic fluctuations in species composition and mean FI, but the overall mean of the FI is unchanging; this suggests that this period was a stable regime characterized by high variability. At ∼4.5 ka species evenness decreases, and the system begins a ∼2 kyr gradual decrease in mean FI. Decreases in FI suggest the system is becoming unstable; as instability increases resilience decreases, warning of a possible regime shift. The system was in this unstable transitional period until ∼2 ka, but it did not attain a new stable-state until ∼1.3 ka.

Fisher information identified a substantial regime shift in the system prior to the abrupt community change. The mean FI results indicated that the system was in a steady state (regime one) from ∼7.0 to ∼4.5 ka. This was followed by a ∼2 kyr period of instability, before it returned to a steady state (regime two) at ∼1.3 ka (Fig. 3). The long period of instability was followed by an abrupt increase in mean FI at ∼2 ka denoting a regime shift [23], which preceded the system regaining stability at ∼1.3 ka, and, thus, returning to a steady state. Regimes one and two are considered stable states, because there is no overall directional trend in mean FI values during those periods [23]. During the ∼2 kyr period of instability, the mean FI decreased steadily, indicating the system was losing dynamic order, and therefore resilience [8]; this slow period of change is a warning of the impending regime shift at ∼2.0 ka.

**Figure 4:**
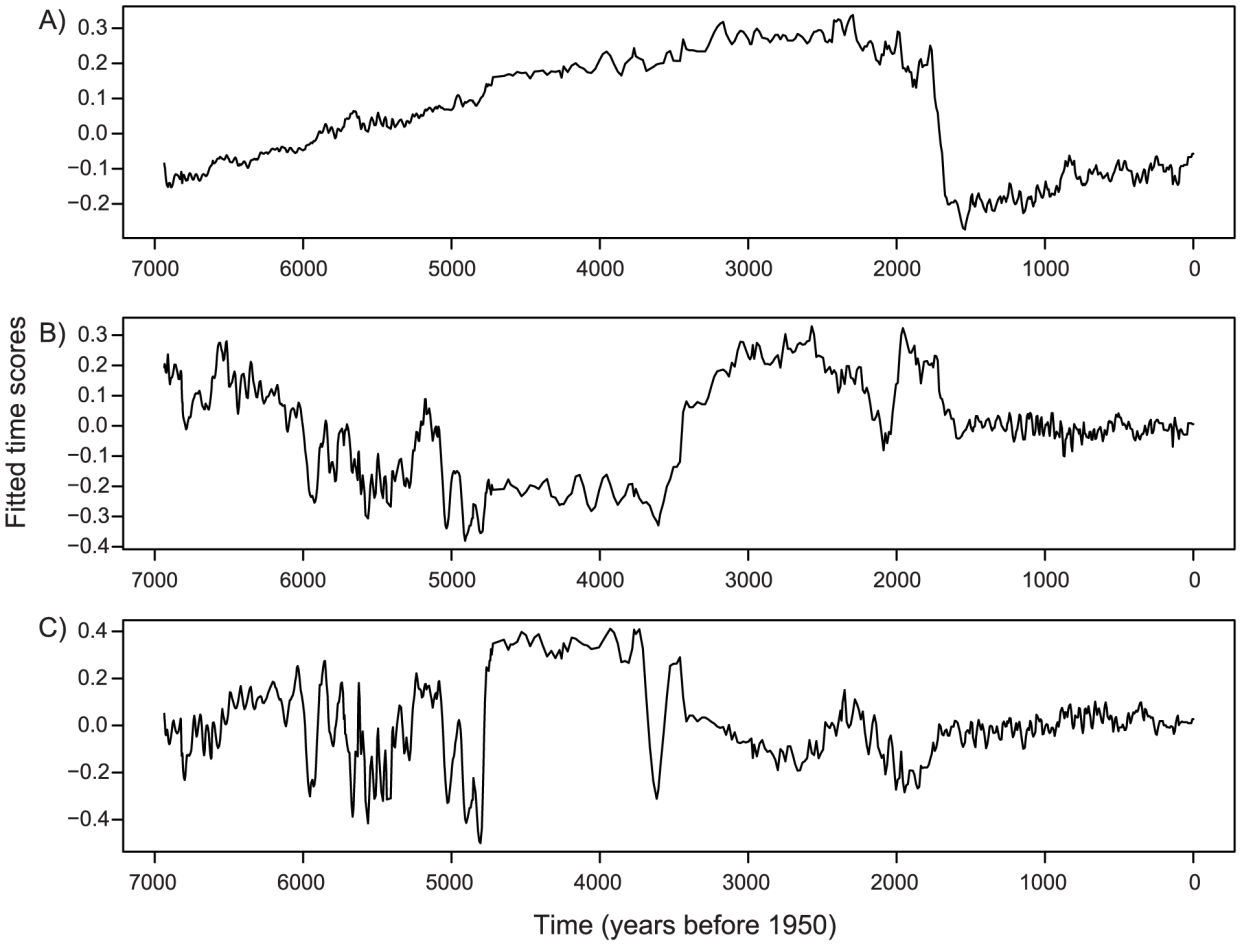
The first three significant axes of the multivariate time-series modeling. The proportion of variance explained by each axis is 29%, 18%, and 8% respectively. The amplitude of the frequency is low in axis one (**A**) with a major shift in score at ∼1.7 ka, indicating a regime shift to an alternate state. This regime shift occurred when the lake changed from a shallow lake dominated by benthic taxa to a deep lake dominated by planktic taxa. Frequency pattern changes are present in axes two (**B**) and three (**C**) at ∼4.8–5 ka and ∼2–1.3 ka, at the beginning and the end of the period of instability.

Multivariate time series modeling revealed eight different temporal patterns in the diatom data set that were associated with eight significant canonical axes in the redundancy analysis (RDA) model. Each of these canonical axes reflects a modeled frequency pattern of individual species or groups of species in the diatom data set. The first three canonical axes capture 55% of the variance used to summarize the transitional dynamics and regime shifts (Fig. 4). The first axis explained the most important pattern in the data set (29% of adjusted variance explained); it separated regime two at ∼1.7 ka from all prior time points (Fig. 4). Axes two and three, which explain 18% and 8% of the variability, respectively, separated the time series into three periods: the first regime from the beginning of the record to ∼4.8 ka, the period of instability that lasts ∼2 kyr, and a second regime that begins at ∼1.7 ka. The frequency patterns in the three axes generated with RDA showed temporal patterns of change that are not exactly the same as those detected in the FI results, but that are complementary. The areas that differ most are the ages of both the onset of instability and of the regime shift; these differences likely occur because FI is a composite of all species, whereas the multivariate analysis partitions species into groups. RDA axis one is a long time interval that includes both regime one and the subsequent transition period between regimes one and two. The major axis break at the onset of regime two suggests that regime two is the stronger of the two stable-states in the system's history. This interpretation is supported by the higher mean FI and lower standard deviation in FI of the second regime (Fig. 4). This pattern was driven by a sudden shift in the relative abundance of diatoms, marked by the onset in numerical dominance of one species (*Cyclotella bodanica* var. *lemanica*) during the second regime (Fig. 1). The transitional period, delineated by mean FI, is not present in the first axis of the time series analysis (Fig. 4). However, it is evident in subsequent axes and reflects gradual changes in species composition and dominance patterns (Fig. 1, 4).

The regimes, transitional period, and regime shift detected by FI and time series modeling are consistent with ecological and regional climate patterns. Foy Lake was a moderately deep lake with a diverse planktic and benthic flora during regime one. Throughout the period of instability, the lake was much shallower and dominated by a benthic flora, and during the more recent regime two, Foy Lake was a deep lake dominated by *Cyclotella bodanica* var. *lemanica*, a planktic species [26]. It is possible that either intrinsic (e.g. nutrients) or extrinsic (e.g. climate change) drivers, or a combination of both are responsible for the abrupt ecological change [27]. However, synchronous change in multiple climate records from the region suggests that extrinsic drivers are likely the cause of the changes to the diatom community structure at Foy Lake. A pattern of recurrent multi-decadal drought in the Foy Lake region ended abruptly ∼4.5 ka [26]; this is at the approximate time that regime one ends and the ∼2 kyr period of instability begins. A shift in the dynamics of the climate system is also evident in multiple other mid-continental paleoclimatic records at ∼4.2 ka [28]. At ∼1.3 ka multiple regional lake records show a synchronous shift in diatom community structure [29], and regular patterns of reoccurring drought returned to the Foy Lake region [30]. Thus, the intervals of recurrent drought on multi-decadal scales coincide with the identified stable regimes in Foy Lake, whereas the onset of the period of instability occurs during a time of persistent severe drought in the mid-continent. There is a lag between the FI identified regime shift and the abrupt change in diatom community structure (from ∼2 ka to 1.3 ka). This lag period is coincident with regional synchronous shifts in diatom communities at multiple lakes at ∼2.2 ka, ∼1.7 ka, and ∼1.35 ka. This suggests that emerging from a period of instability may involve several smaller short-lived transitions in ecosystem state before long term stability is achieved.

Paleoenvironmental and paleoecological data provide a vital and fundamental perspective on the long-term functioning of complex ecological systems. Here we reveal that climate-driven regime shifts may be infrequent over time in systems not impacted by anthropogenic change, and that transitional periods leading to a regime shift can last a relatively long time (∼2.0 kyr). Delayed responses and time lags have been found in other ecosystems [31]–[33], and these may provide a false sense that the ecosystems are stable, leading to their mismanagement [34]. It is likely that some ecosystems are currently in prolonged periods of instability, whereby they are losing resilience and are exposed to compounding stresses driven by anthropogenic change. Moreover, when disturbance is large-scale and long-term, some early-warning signals may occur long before the system settles into an alternate stable regime, and the lag between signal and stability may be difficult to predict. Here we suggest effective tools (FI and multivariate time series modeling) to detect and understand changes in those ecosystems that are susceptible to periods of prolonged instability prior to regime shifts.

## Methods

### Calculating Early Warning Signals

Rising variance, skewness, kurtosis, and critical slowing down are statistical measures that have been proposed and employed as indicators of impending regime shifts [11], [16], [35]–[37]. Most of the indicators (i.e., variance, skewness, and kurtosis) are straightforward and can be computed using readily available functions in standard statistical packages (e.g., the Matlab function for computing variance is var). Critical slowing down is estimated by using the lag-1 autocorrelation coefficient [11]. Hence, the autocorrelation function is used to calculate this indicator. For the sake of consistency, all statistical indicators were computed from the percent abundance of each diatom species given the same window size (10 time steps) over the 7 kyr record using Matlab (Release 2012a, Mathworks, Inc.).

### Fisher Information

Fisher information (FI) can be used to evaluate the dynamic order of ecosystems, including regimes and regime shifts [23]–[24]. Unlike early warning signals, FI characterizes changes in complex system dynamics as a function of patterns in underlying variables (e.g., species abundances of diatoms) by collapsing their behavior of into an index that can be tracked over time [23]. The form of Fisher information (*I*) used in this work was adapted by Fath et al. [18] and Mayer et al. [38]. 
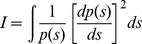
(1)


Here, p(s) is the probability of observing the system in a particular condition (state, s) of the system. This equation was adapted [17]–[18], such that FI could be computed analytically or estimated numerically [23]. The numerical approach of FI was applied in this work and calculated from the following expression (derived in detail by Karunanithi et al. [23]: 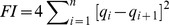
(2)where, the probability density p(s) is replaced by its amplitude (q^2^(s)≡p(s)) in order to minimize calculation errors from very small p(s). From Equation 1, note that FI is proportional to the change in the probability of a system being in a particular state (*p(s)*) versus the change in state *ds*, i.e., *FI*∝*dp*/*ds* [18].

### Calculating FI

Assessing the dynamic changes in system behavior requires gathering information on its condition (state) through time; hence, measurable variables (x_i_) are selected such that a time varying system has a trajectory in a phase space defined by the n-dimensions of its system variables and time. Each point in the trajectory is defined by specific values for each of the *n* variables (i.e., a point at time *i* is defined as [x_1_(t_i_), x_2_(t_i_) x_3_(t_i_)…x_n_ (t_i_)]). Since uncertainty is inherent in any measurement and system variables may fluctuate within a stable state, a state is defined as a region bounded by a level of uncertainty (or size of states for each dimension (i): sost_i_), such that if |x_i_(t_i_) - x_i_ (t_j_)| ≤ sost_i_ is true for all variables then the two points at times i and j are indistinguishable and are identified as being in the same state of the system. There are a number of methods for defining the sizes of states parameter, but the general idea is to assign a level of uncertainty for each variable based on either knowledge of the system (empirically or theoretically) or estimation [24]. Given this conceptual description of systems and states, the probability *p*(*s*) of a system being in a particular state (*s*) can be estimated by counting the number of observational data points that meet the size of states criteria. Using this approach, it is possible to designate all possible states of the system over time.

The basic steps employed to compute FI for the Foy Lake system were as follows: (1) the diatom time series data (consisting of the relative abundances of all 109 species) were divided into a sequence of overlapping time windows with each window containing 10 time steps. Since the goal is to capture changing patterns, there is no particular window size that must be used to compute FI. The window size is set based on available data and from empirical studies, it is typically at least eight time steps [39]. (2) The level of uncertainty was estimated by searching for the window (i.e., 10 time steps) within the diatom time series with the least amount of variability. The standard deviation for each species was then calculated to establish the size of states criteria and bin points into states. (3) The binned points were then used to generate probability densities, *p(s)*, for each state. (4) Equation 2 was used to compute a unique FI for each window resulting in a sequence of FI values over time. The algorithm for computing FI was coded in Matlab (Release 2012a, Mathworks, Inc). Additional details of the FI derivation, calculation methodology, and computer code can be found in [23], [39].

### Interpreting FI

Assessing system behavior using FI is based on the fundamental idea that different regimes (set of system conditions) exhibit different degrees of dynamic order [23]. In practical terms, a regime fluctuates within a range of variation, such that the overall condition does not change from one observation to another. Hence, the resulting FI is non-zero and remains relatively stable through time. Steadily decreasing FI signifies loss of dynamic order and resilience of a regime and provides warning of an impending regime shift. A decrease in FI between two stable dynamic regimes denotes a regime shift [8], [23]. This shift point is typically identified as a minimum FI value after which FI will often increase. While steadily rising FI is indicative of increasing dynamic order, it denotes a shift to a new regime, only if the increase is followed by a new stable regime (i.e., period in which *d*〈*FI*〉/*dt*≈0). Note that there is no guarantee that the latter regime is more desirable than the former, i.e., while the condition of the system may be stable, the system could have organized into a less desirable regime (e.g., eutrophic lake). Hence, FI affords the ability to assess the stability of a system, not the quality of its condition [25]. Further evaluation of the underlying variables is required to determine whether the system state is desirable.

### Multivariate time series modeling

To assess patterns and scales of diatom fluctuations, we constructed time series models based on redundancy analysis (RDA) [9], and used temporal variables extracted by PCNM (Principal Coordinates of Neighbor Matrices) analysis [40]–[41]. Briefly, the PCNM analysis converts the linear time vector that comprises the sampling frequency and length of the study period into a set of orthogonal temporal variables. In our study, the time vector consisted of 800 time steps during the 7 kyr study period. The PCNM analysis yielded 517 variables with sine-wave properties from the conversion of the linear time vector. Each PCNM variable corresponds to a specific temporal frequency in the diatom dynamics. That is, the first PCNM variable models the longest temporal frequency while the subsequent variables capture temporal variability from longer to increasingly shorter fluctuation frequencies in the community data over the study period. We constructed a parsimonious RDA model for diatom community dynamics by running a forward selection on the 517 PCNM variables.

The RDA retains significant PCNM variables, and these are linearly combined to extract temporal patterns from the Hellinger-transformed species matrices [42]; that is, the RDA identifies species with similar temporal patterns in the species × time matrix and uses their temporal patterns to calculate a modeled species group trend for these species based on linearly combined PCNMs. The significance of the temporal patterns of all modeled fluctuation patterns of species groups revealed by the RDA is tested by means of permutation tests. The RDA relates each modeled temporal fluctuation pattern with a significant canonical axis. The R software generates linear combination (lc) score plots, which visually present the modeled fit of temporal patterns of species groups that are associated with each canonical axis. Because the canonical axes are orthogonal (independent from each other), one can assess the number of temporal scales at which community dynamics unfold. All relevant steps in the time series analysis are carried out using the “quickPCNM” function in R 2.15.0 (R Development Core Team).

## Supporting Information

Dataset S1 **Percent abundances of diatom species from Foy Lake calculated relative to the total number of diatom valves counted in each sample.** Time steps with no diatom data, due to poor preservation, were removed from the dataset. Time steps 301–312 were averaged for these analyses, because they were assigned the same age, as per the age model.
(CSV)
